# LINC01605, regulated by the EP300-SMYD2 complex, potentiates the binding between METTL3 and SPTBN2 in colorectal cancer

**DOI:** 10.1186/s12935-021-02180-8

**Published:** 2021-09-20

**Authors:** Meng Yue, Tao Liu, Guoqiang Yan, Xiaofan Luo, Lei Wang

**Affiliations:** Department of Colorecal and Anal Surgery, The First Hospital of Jilin University, No. 71, Xinmin Street, Chaoyang District, Changchun, 130021 Jilin People’s Republic of China

**Keywords:** H3K4me3, H3K27ac, LINC01605, m6A, SPTBN2, METTL3, Colorectal cancer

## Abstract

**Background:**

Colorectal cancer (CC) is one of the major contributors to tumor-related death worldwide, and its main cause of death is distant metastasis. Dysregulation of long non-coding RNA (lncRNA) LINC01605 has been implicated in CC. However, its role in metastasis of CC remains elusive. The goal of the study is to uncover the biological function and molecular mechanism of LINC01605 in CC.

**Methods:**

The differentially expressed lncRNAs were first screened from GSE97300, GSE84983, GSE110715, GSE70880, and GSE75970 microarrays. The correlation between the expression of LINC01605 and the clinical phenotypes of enrolled CC patients (n = 134) was subsequently analyzed. The upstream and downstream regulatory mechanisms of LINC01605 in CC were identified through bioinformatics and RNA-seq analyses. Finally, the effects of related factors on CC cell growth and metastasis were confirmed through functional validation experiments.

**Results:**

LINC01605, significantly highly expressed in CC, was a prognostic factor for patients with CC. Functional experiments revealed that LINC01605 knockdown inhibited the proliferatory and metastatic potential of CC cells in vitro and in vivo. Moreover, LINC01605 was regulated by SMYD2-EP300-mediated modifications of histone H3K4me3 as well as H3K27ac. LINC01605 was found to bind to METTL3 and promote the m6A modification of SPTBN2 mRNA, thereby facilitating the translation of SPTBN2.

**Conclusions:**

Overexpression of LINC01605, regulated by SMYD2-EP300-mediated H3K27ac and H3K4me3 modifications, bound to METTL3 protein to promote m6A modification of SPTBN2 mRNA, leading to the development of CC.

**Supplementary Information:**

The online version contains supplementary material available at 10.1186/s12935-021-02180-8.

## Background

More than 104,270 colorectal cancer (CC) diagnoses and more than 52,980 deaths from the disease are predicted in 2021 in the United States [1]. The cornerstones of therapy for CC include surgery, neoadjuvant radiotherapy, and adjuvant chemotherapy (for patients with advanced stages), and 5-year relative survival varies from greater than 90% in patients at stage I to slightly greater than 10% in patients at stage IV [2]. Therefore, exploring the mechanisms underlying CC progression will facilitate the search for the new diagnostic markers and the development of applicable therapeutic targets.

In the epigenetic mechanisms, long non-coding RNAs (lncRNAs) are identified as significant contributors to the initiation, progression and metastasis of CC [3]. For one of them, LINC01605 was upregulated in 31 out of 38 cases of CC tissues, and its expression in CC cells was higher than that in normal colorectal cells [4]. Therefore, we set to determine the specific role as well as the underlying mechanism of LINC01605 in CC. A previous genome-wide analysis of histone modifications in CC cells revealed that the transcription start sites of 1027 lncRNAs acquired trimethylation of histone H3 lysine 4 (H3K4me3) following DNA demethylation [5]. Histone H3 on lysine 27 acetylation (H3K27ac), another class of histone posttranslational modification, is frequently linked to the active enhancer regulatory elements, contributing to the upregulation of genes [6]. For instance, lncRNA GHET1 activated by H3K27ac promoted tumorigenesis in hepatocellular carcinoma [7]. Our preliminary prediction, intriguingly, revealed significant H3K4me3 and H3K27ac modifications near the promoter of LINC01605. Therefore, we postulated that the upregulation of LINC01605 was due to the H3K4me3 and H3K27ac modifications.

N6-methyladenosine (m6A), a chemical modification existing in manifold RNA species, is installed by a multicomponent methyltransferase complex, including methyltransferase-like 3 (METTL3), METTL Wilms tumor 1 associated protein, KIAA1429, RNA binding motif protein 15, and zinc finger CCCH domain-containing protein 13 [8]. A previous methylated RNA immunoprecipitation sequencing (MeRIP-seq) identified spectrin beta, non-erythrocytic 2 (SPTBN2) as one of the four genes with differentially methylated m6A peaks and differential expression in CC patients in The Cancer Genome Atlas (TCGA) database [9]. Interestingly, LINC00460 has been reported to regulate HMGA1 expression depending on METTL3-modulated m6A modification of HMGA1 mRNA [10]. Considering these reports, it is reasonable to assume that overexpression of LINC01605, mediated through H3K4me3 and H3K27ac modifications, could enhance the expression of SPTBN2 in a METTL3-dependent m6A modification in CC. The present study proposed to uncover the function of LINC01605 in CC, and the downstream and upstream mechanisms of action.

## Methods

### Patients and tissue samples

A total of 134 CC patients (71 males and 63 females, age 39–72 years, mean age 52.8 ± 7.2 years) admitted to the First Hospital of Jilin University from July 2014 to March 2017 were included in this study. All patients were diagnosed by histopathological examination. Inclusion criteria were as follows: (1) newly diagnosed cases; (2) no treatment prior to the admission; and (3) patients who signed an informed consent form. Patients who were transferred from other institutions, had concurrent diseases other than CC, or received treatments were excluded. All patients were staged on the basis the criteria proposed by the American Joint Committee on Cancer. This study was carried out following the *Declaration of Helsinki*, and permitted by the Ethics Review Committee of the First Hospital of Jilin University.

### Analysis of public databases

First, the CC-related gene expression microarrays GSE97300 (containing four cases of CC tissue and four cases of normal colon tissues), GSE84983 (containing cancer tissue and paracancerous tissue from six CC patients), GSE110715 (containing cancer tissue and paracancerous tissue from six CC patients), GSE70880 (containing cancer tissue and paracancerous tissue from 20 CC patients), and GSE75970 (containing cancerous and paracancerous tissues from four CC patients were downloaded). Variance analysis was performed using the R Limma package with screening thresholds set at Log FoldChange > 2.0 or < − 2.0 and adj. *p* value < 0.05, and heat maps were plotted using ggplot2.

Subsequently, chromatin immunoprecipitation (ChIP)-sequencing (seq) data of normal colon tissues and colon cancer tissues were downloaded from the GEO database (GSE36204) which contains ChIP-seq data of H3K4me1, H3K4me3, and H3K27ac. Data analysis was conducted using Bowtie 2, and the results were visualized using the IGV software.

### mRNA and protein expression assays

We used RT-qPCR, western blot and immunohistochemistry to detect the expression of mRNA and protein in cells and tissues. Details are provided in the Additional file 1.

### Cells and culture conditions

Human Caco-2 and Lovo cells were from ATCC (Manassas, VA, USA). These cells were tested free of mycoplasma and cultured in Ham’s F-12 K (Kaighn’s) medium (Gibco, Carlsbad, CA, USA) or RPMI-1640 medium (Hyclone, Marlborough, MA, USA) plus 10% heat-inactivated FBS (Gibco), 100 μg/mL streptomycin and 100 U/mL penicillin (Gibco) in 5% CO_2_ at 37 °C.

Plasmid-mediated SPTBN2 (NM_006946) vector, SET and MYND domain-containing protein 2 (SMYD2) vector (NM_020197), and E1A binding protein p300 (EP300) vector (NM_001409), and knockout vectors for LINC01605 or METTL3 (NM_019852) were from Genechem (Shanghai, China), and empty vectors served as a negative control (NC).

Subsequently, Caco-2 and Lovo cells in good growth condition were analyzed using colony formation and CCK-8 assays for cell growth and proliferation activity, flow cytometry and TUNEL staining for apoptosis, Transwell assay for migration and invasion, and tube formation assay for the angiogenesis of human umbilical vein endothelial cells (HUVECs). Details are provided in the Additional file 1.

### Animal experiments

All animal assay processes were permitted by the Ethics Committee of the First Hospital of Jilin University for the use of animals and conducted in accordance with the NIH Laboratory Animal Care and Use Guidelines. Every effort was made to minimize the pain of mice. The growth of CC cells in vivo was examined by subcutaneous injection of LoVo cells or Caco-2 cells into NSG mice, while the metastatic ability of cells in vivo by intracardiac injection into mice, as detailed in the Additional file 1.

### ChIP-qPCR

Briefly, paired tumor tissues as well as paracancerous tissues were treated with lysis buffer containing proteinase inhibitors (Roche, Indianapolis, IN, USA), and then chromatin was ultrasonically fragmented. Then, the lysate (20 μg) was immunoprecipitated with 2 μg antibodies to H3K27ac (ab4729, Abcam), H3K4me3 (ab8580, Abcam) using an EZ-Magna ChIP™ G kit (Millipore, Temecula, CA, USA) or mouse antibody IgG (1 μg, ab37406, Abcam). Quantification of IP-DNAs was implemented with a LightCycler 480 system Real-TiME PCR instrument (Roche) and RealQ Plus 2 × Master Mix (Ampliqon, Denmark). All oligonucleotides were designed with the Primer3Plus program and synthesized by Sangon (Shanghai, China).

### Fluorescence co-localization

The cell slides were incubated overnight at 4 °C with the primary antibody to EP300 (ab275378, 1:200, Abcam), followed by incubation with secondary antibody Cy3-conjugated donkey anti-rabbit IgG (C2571, 1:100; Sigma-Aldrich) for 120 min at ambient temperature. After that, the slides were incubated with the antibody to SMYD2 (ab234862, 1:200, Abcam) for 4 h at room temperature, followed by incubation with secondary antibody FITC-conjugated donkey anti-goat IgG (F3512, 1:100; Sigma-Aldrich) for 2 h at room temperature. After nuclei counter-staining with DAPI (Vector Laboratories, Burlingame, CA, USA), fluorescent mounting medium (Dako Cytomation, Glostrop, Denmark) was used to mount the slides. All images were taken under a confocal microscope (LSM FV 1000; Olympus).

### Luciferase assay

The wild-type SPTBN2 CDS (SPTBN2) fragment containing the predicted METTL3 target site was amplified by PCR and subcloned into the pGL3 vector (Promega), which included the firefly luciferase reporter gene. Wild-type METTL3 (METTL3-WT) was PCR-amplified and cloned into the pcDNA3.1 vector, which included the firefly luciferase reporter gene. The binding domain of METTL3 was then mutated using a gene mutation kit (Takara Biotechnology Ltd., Dalian, Liaoning, China) to generate mutant METTL3 (METTL3-MT). Then, the pRL-TK vectors (Promega) containing the Renilla luciferase reporter gene, the METTL3-MT or METTL3-WT plasmids, and SPTBN2 were co-transfected into LoVo or Caco-2 cells. A negative control was set using the empty pcDNA3.1 vector. After 2 days, firefly and Renilla luciferase activities were determined using a dual luciferase reporter system (Promega) with Renilla luciferase activity for normalization.

### CRISPR-Cas9 system

The sgRNAs for deleting LINC01605 enhancers were designed using CRISPRscan (https://www.crisprscan.org) and CRISPRdirect (http://crispr.dbcls.jp) (sgRNA-1 GGTAGGGCGACTGCGTTCAA; sgRNA-2 GGGCCCGGCCCGAGCGCTAA). sgRNAs were annealed with NEBuffer2 at 95 °C for 5 min and at 70 °C for 10 min. With the help of BbsI and BsaI (New England Biolabs, Ipswich, MA, USA), double-stranded DNA was inserted into the CRISPR/Cas9 PX458 vectors. The purified recombinant plasmids were transfected into the Caco-2 and LoVo cells in a 24-well plate. After 48 h, puromycin screening was conducted, and the cells were separated into 96-well plates by limiting dilution. PCR was amplified from the DNA isolated from homozygous (SE^−/−^) clones using primers of LINC01605-enhancer. The knockout plasmids of enhancer were transfected for 2 days and treated with appropriate puromycin for 3 days. The cells were collected to test the knockout efficiency and gene expression.

### Microarray sequencing

Total RNA from CC cells with LINC01605 knockdown was extracted with TRIzol reagent (Takara) and quantified with NanoDrop ND-1000 (Thermo Fisher Scientific). The sample preparation and microarray mixing were implemented by Aksomics (Shanghai, China), followed by the standard Arraystar protocol. Microarray analysis of RNA was achieved by sample preparation on NuRNA™ mRNA PCR arrays.

### RNA immunoprecipitation (RIP)

The EZ-Magna RIP kit (Millipore) was used. First, the cells are lysed using RIP lysis buffer with RNase and protease inhibitors (Millipore). Second, the RIP lysate was subjected to RIP buffer containing magnetic beads conjugated with mouse anti-human to METTL3 (ab195352, Abcam) or non-specific IgG antibody (ab37406, Abcam). Next, immunoprecipitates were detached using proteinase K. RNA concentration was measured by NanoDrop spectrophotometer, and purified RNA was analyzed using RT-qPCR.

### Biotin-label RNA pull-down

RiboBio (Guangzhou, Guangdong, China) was commissioned to synthesize biotinylated-LINC01605 or SPTBN2 mRNA probes. Lovo or Caco-2 cells (1 × 10^7^) were lysed and cultured with biotinylated-LINC1605 or SPTBN2 mRNA probes. Next, biotin-coupled RNA complexes were pulled-down using streptavidin-coated magnetic beads. The enriched METTL3 was determined using western blot.

### MeRIP-seq and MeRIP-qPCR

Total RNA was isolated from Lovo and Caco-2 cells transfected with sh-LINC01605 or sh-NC using TRIzol (Invitrogen Inc., Carlsbad, CA, USA). Complete mRNA was obtained using the Seq-Star™ poly(A) mRNA Isolation Kit (Arraystar Inc., Rockville, MD, USA). After fragmentation, the RNA (100 nucleotides) was probed with m6A antibody (ABE572, Merck Millipore, Darmstadt, Germany) according to the protocols provided by the MeRIP m6A kit (Merck Millipore). The mRNAs with m6A enrichments were then detected using NGS or RT-qPCR. For NGS, RNA fragments were purified from m6A-MeRIP and sequenced with Illumina HiSeq X-10 after library construction with Illumina’s (New England Biolabs) NEBNext Ultra RNA library Prep kit. The library construction and NGS were done by Aksomics. FastQC (v0.11.7) was used for quality inspection of raw sequencing data, and original sequence was filtered using Trimmomatic (V0.32). Filtered high-quality data were compared with the reference genome (HISAT2 v2.1.0) in the Ensembl database. ExomePeak (v2.13.2) was used to identify peaks in each sample and to identify differentially methylated peaks in compared samples. Peaks were annotated according to Ensembl database annotation information, and peaks in different regions [5′ untranslated region (5′UTR), coding sequences (CDS), and 3′ untranslated region (3′UTR)] of each transcript were counted in every sample. MeRIP-qPCR was performed to measure the m6A levels of SPTBN2 in CC cells. Primers targeting the m6A negative/positive site of MAP2K4 served as negative/positive controls.

### Statistics

Experiments were performed in triplicate. Data were presented as mean ± standard deviation (SD). SPSS Statistics 22 software (IBM Corp. Armonk, N.Y., USA) and GraphPad Prism 8.0 (GraphPad, San Diego, CA, USA) were used for statistical analyses. Spearman’s correlation coefficient was used to calculate the correlations between two groups. Kaplan–Meier method and log-rank test were utilized for survival analysis. Comparisons between two groups were performed using an unpaired or paired *t* test. Differences among the groups were compared by one-way or two-way ANOVA, followed by Tukey’s post hoc test. *p* < 0.05 was considered to reflect a statistically significant difference.

## Results

### LINC01605 is overexpressed in patients with CC

**Fig. 1:**
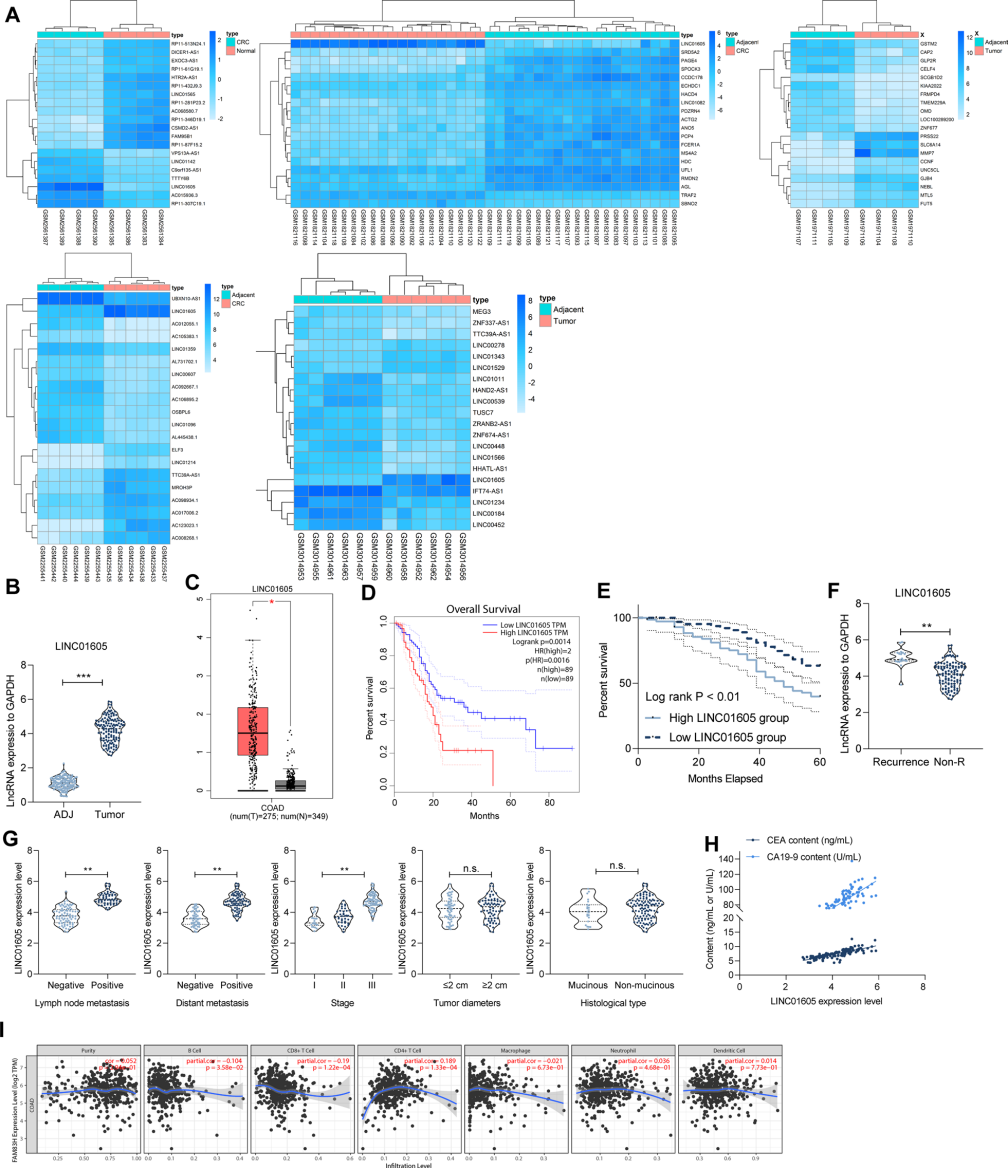
LINC01605 is highly expressed in patients with CC. **A** the heatmap of the top 20 differentially expressed lncRNAs in GSE97300, GSE84983, GSE110715 and GSE70880, GSE75970 gene expression microarrays. **B** expression of LINC01605 in cancer and ADJ tissues of 134 CC patients were detected by RT-qPCR. **C** expression of LINC01605 in the TCGA-COAD database. **D** Kaplan–Meier analysis of the correlation between the expression of LINC01605 and the survival of COAD patients. **E** Kaplan–Meier analysis of the correlation between the expression of LINC01605 and the survival rate of 134 CC patients. **F** LINC01605 expression difference in tumor tissues of patients with recurrence or not measured using RT-qPCR. **G** analysis of the correlation between the expression of LINC01605 and the clinical phenotype of 134 patients. **H** Spearman’s correlation analysis of LINC01605 expression with the levels of CC markers CEA and CA199 in patients’ serum. **I** Cibersort analysis of the correlation between LINC01605 expression and immune cell infiltration in tumor tissues of COAD patients. Error bars represent means ± SD for three independent experiments (*p < 0.05, **p < 0.01 and ***p < 0.001, ns, not significant. paired or unpaired *t* test, one-way ANOVA, followed by Tukey’s multiple comparison)

We screened differentially expressed genes from GSE97300, GSE84983, GSE110715, GSE70880, and GSE75970, separately (Fig. 1A), and identified seven intersecting genes, LINC01605, ZNF337-AS1, LINC01082, DICER1-AS1, LUCAT1, MEG9, and SNHG20. Therefore, we examined their expression in our collected cancer tissues and adjacent (ADJ) tissues from 134 CC patients. LINC01605, SNHG20, LUCAT1, and ZNF337-AS1 had significantly high expression in tumor tissues, while MEG9, LINC01082, and DICER1-AS1 had high expression in ADJ tissues (Fig. 1B, Additional file 2: Figure S1A). We further queried the expression of these differentially expressed genes from TCGA-colon adenocarcinoma (COAD) database and analyzed their connection to the prognosis of the patients. It was found that LINC01605 was significantly highly expressed in COAD patients and was associated with a poor prognosis (Fig. 1C, D and Additional file 2: Figure S1B, C).

Moreover, we followed-up these 134 CC patients for a period of 36–60 months, and divided the patients into high LINC01605 group and low LINC01605 group according to the expression of LINC01605. Kaplan–Meier analysis of the association between the expression of LINC01605 and the survival rate of patients revealed that patients with high expression of LINC01605 had shorter survival time (Fig. 1E). And then, 19 of 134 patients had recurrence after surgery, and we noticed that the LINC01605 expression was much higher in these 19 patients than that in patients with non-recurrence (Non-R) (Fig. 1F). In the subsequent analysis, we detailed the correlation between the patients’ clinical phenotypes and LINC01605 expression. The results showed that CC patients with high LINC01605 expression in tumor tissues inclined to have lymph node metastases, distal metastases, and advanced tumor stage. However, its expression was not related to the diameter and histological type of tumor tissues (Fig. 1G). Moreover, we further found that the serum levels of CC-related markers CEA and CA19-9 were positively correlated with LINC01605 expression in 134 patients (Fig. 1H). The expression of LINC01605 had a significant positive correlation with immune cell infiltration in the tumor tissues of COAD patients in the TCGA database (Fig. 1I). Based on these results, we believe that overexpression of LINC01605 contributed to the development and progression of CC.

### LINC01605 is regulated by the SMYD2-EP300 complex

To understand why LINC01605 is highly expressed in CC tissues, we first searched for cis-regulatory elements upstream of LINC01605 using the UCSC browser website (http://genome.ucsc.edu/index.html). We found significant H3K27ac and H3K4me3 modifications near the LINC01605 promoter (Additional file 3: Figure S2A). To verify our conjecture, we further downloaded ChIP-seq data (GSE36204) from the GEO database for normal colon tissues and CC tissues, which contained data for three super enhancer (SE)-like signatures, H3K4me1, H3K4me3, and H3K27ac. The results established that the number of H3K4me1-, H3K4me3-, and H3K27ac-modified genes was much higher in CC tissues than that in normal colon tissues (Additional file 3: Figure S2B). Moreover, we further visualized the data. Interestingly, the modification levels of H3K4me1, H3K4me3, and H3K27ac near the LINC01605 promoter were significantly promoted in CC tissues, and the modification levels of H3K4me3 as well as H3K27ac were more pronounced than those of H3K4me1 (Additional file 2: Figure S2C).

**Fig. 2:**
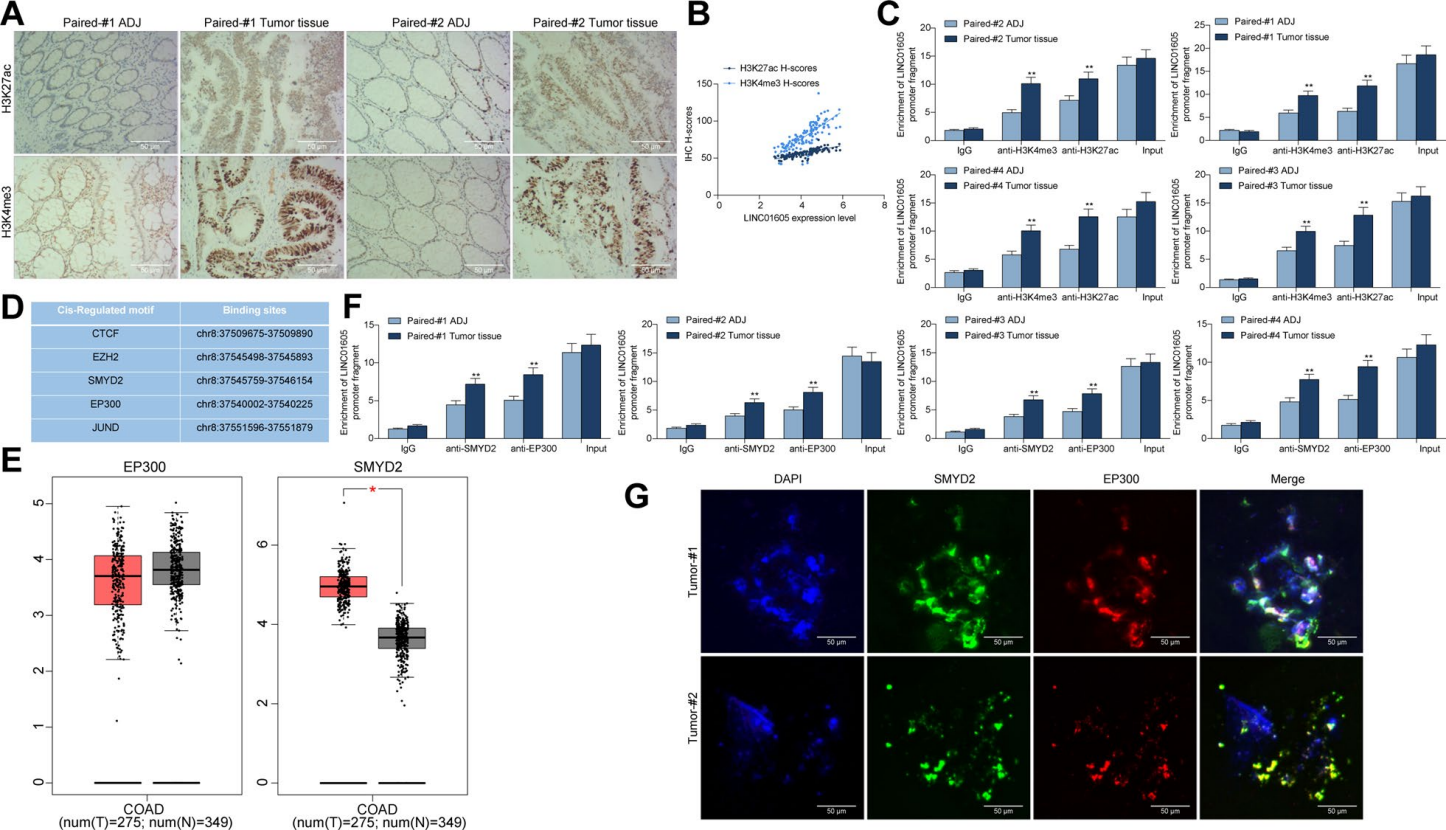
LINC01605 is regulated by the SMYD2-EP300 complex. **A** staining of H3K4me3 and H3K27ac in cancer and ADJ tissues of CC patients examined using immunohistochemistry. **B** Spearman’s correlation analysis of the staining intensity of H3K27ac or H3K4me3 with the expression of LINC01605 in CC tissues. **C** H3K27ac or H3K4me3 recruitment levels of LINC01605 promoter in tumor and ADJ tissues of four CC patients measured using ChIP-qPCR. **D** R TFBS package and the JASPAR website prediction of regulatory factors that bind to the LINC01605 promoter. **E** expression of SMYD2 and EP300 in TCGA-COAD database; **F** SMYD2 or EP300 recruitment levels of LINC01605 promoter in tumor and ADJ tissues of four CC patients examined using ChIP-qPCR; **G** the subcellular localization of SMYD2 and EP300 in CC tissues detected using fluorescence co-localization assay. Error bars represent means ± SD for three independent experiments (**p < 0.01, two-way ANOVA, followed by Tukey’s multiple comparison)

Thus, we examined the staining intensity of H3K4me3 and H3K27ac in tumor and ADJ tissues of 134 CC patients using immunohistochemistry. Their staining intensities in tumor tissues were significantly higher than that in ADJ tissues, and the staining H-scores was positively correlated with the LINC01605 expression in tumor tissues (Fig. 2A, B). We analyzed the H3K27ac or H3K4me3 recruitment levels of LINC01605 promoter in tumor tissues and ADJ tissues of four CC patients using ChIP-qPCR. LINC01605 promoter had more modification levels of H3K27ac and H3K4me3 in tumor tissues (Fig. 2C).

To further refine its possible upstream mechanism, we used the R TFBS package as well as the JASPAR website (http://jaspar.genereg.net/) to predict the regulatory factors that can bind to the LINC01605 promoter, and we found that both SMYD2 and EP300 can bind to the LINC01605 promoter (Fig. 2D). It was worthwhile to note that SMYD2 was significantly highly expressed in CC patients in the COAD database, while EP300 was not differently expressed in TCGA-COAD database (Fig. 2E). However, we further used antibodies targeting EP300 or SMYD2 to detect the enrichment of LINC01605 in the four patients. The enrichment of EP300 or SMYD2 in the promoter region of LINC01605 were much higher in cancer tissues than that in ADJ tissues (Fig. 2F). We then confirmed the subcellular localization of EP300 and SMYD2 in cancer tissues using fluorescence co-localization experiments, and we found that SMYD2 had co-localization with EP300 in the nucleus in cancer tissues (Fig. 2G). The above results can tentatively suggest that aberrant H3K4me3 and H3K27ac modifications lead to high expression of LINC01605 and thus to the development and progression of CC, but the exact mechanism needs to be further investigated.

### Knockdown of LINC01605 impairs the colony formation of CC cells

**Fig. 3:**
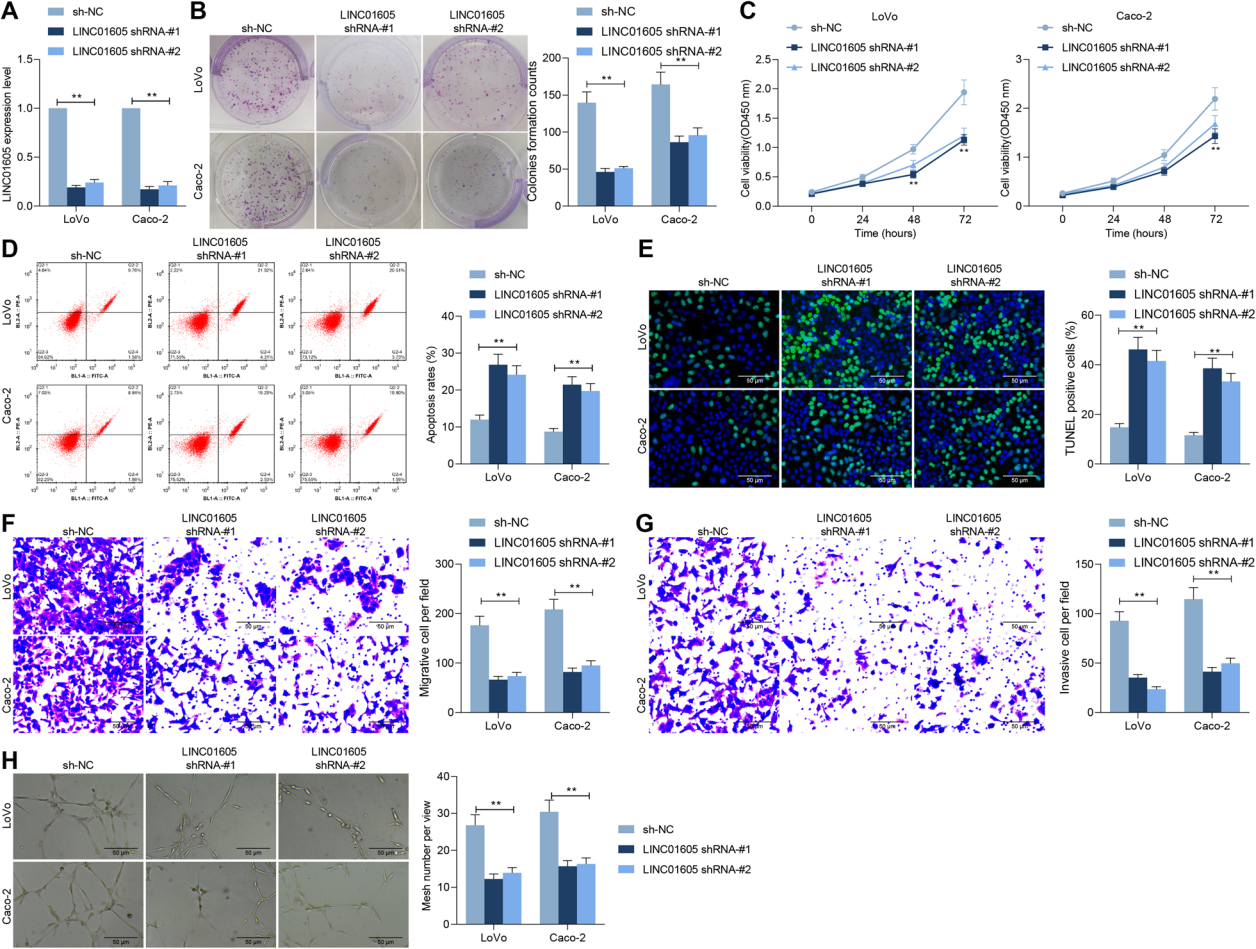
Knockdown of LINC01605 impairs the colony formation of CC cells. ShRNAs targeting LINC01605 (shRNA-1 and shRNA-2) were transfected into LoVo and Caco-2 cells. **A** LINC01605 expression in cells measured by RT-qPCR. **B** the number of colonies formed by LoVo and Caco-2 examined using colony formation assay. **C** the proliferative activity of LoVo and Caco-2 examined using CCK-8 assay. **D** the proportion of apoptotic cells in LoVo and Caco-2 cells measured using flow cytometry. **E** the number of apoptosis bodies in cells examined using TUNEL assay. **F**, **G** the altered migration and invasion ability of LoVo and Caco-2 cells measured using Transwell assay. **H** the number of tubes formed by HUVEC cells co-cultured with Caco-2 and LoVo cells evaluated using tube formation assay. Error bars represent means ± SD for three independent experiments (**p < 0.01, two-way ANOVA, followed by Tukey’s multiple comparison)

To verify the mechanism of action of LINC01605 in CC growth and metastasis, we first transfected shRNAs targeting LINC01605 (shRNA-#1 and shRNA-#2) into LoVo and Caco-2 cells and confirmed the transfection efficiency by RT-qPCR (Fig. 3A). The number of colonies formed by LoVo and Caco-2 cells was drastically decreased in the presence of shRNAs (Fig. 3B), and the proliferative activity of the cells was also significantly downregulated (Fig. 3C). Subsequently, we analyzed the apoptotic rates of cells, and we found a significant increase in the apoptotic rates of LoVo and Caco-2 cells after knockdown of LINC01605 (Fig. 3D) and a significant augment in the number of apoptosis bodies (Fig. 3E). And, we found a significant decline in the migration and invasion in cells with low expression of LINC01605 by Transwell assays (Fig. 3F, G). To further determine the regulatory role of LINC01605 in CC, we established an experimental model of angiogenesis, and we observed a significant reduction in the number of HUVEC-forming vessels induced in Caco-2 and LoVo cells transfected with shRNAs (Fig. 3H). The above results can tentatively indicate a significant correlation between LINC01605 expression and the growth and metastasis of CC cells in vitro.

### Knockdown of LINC01605 impairs the growth and metastatic activity of CC cells

**Fig. 4:**
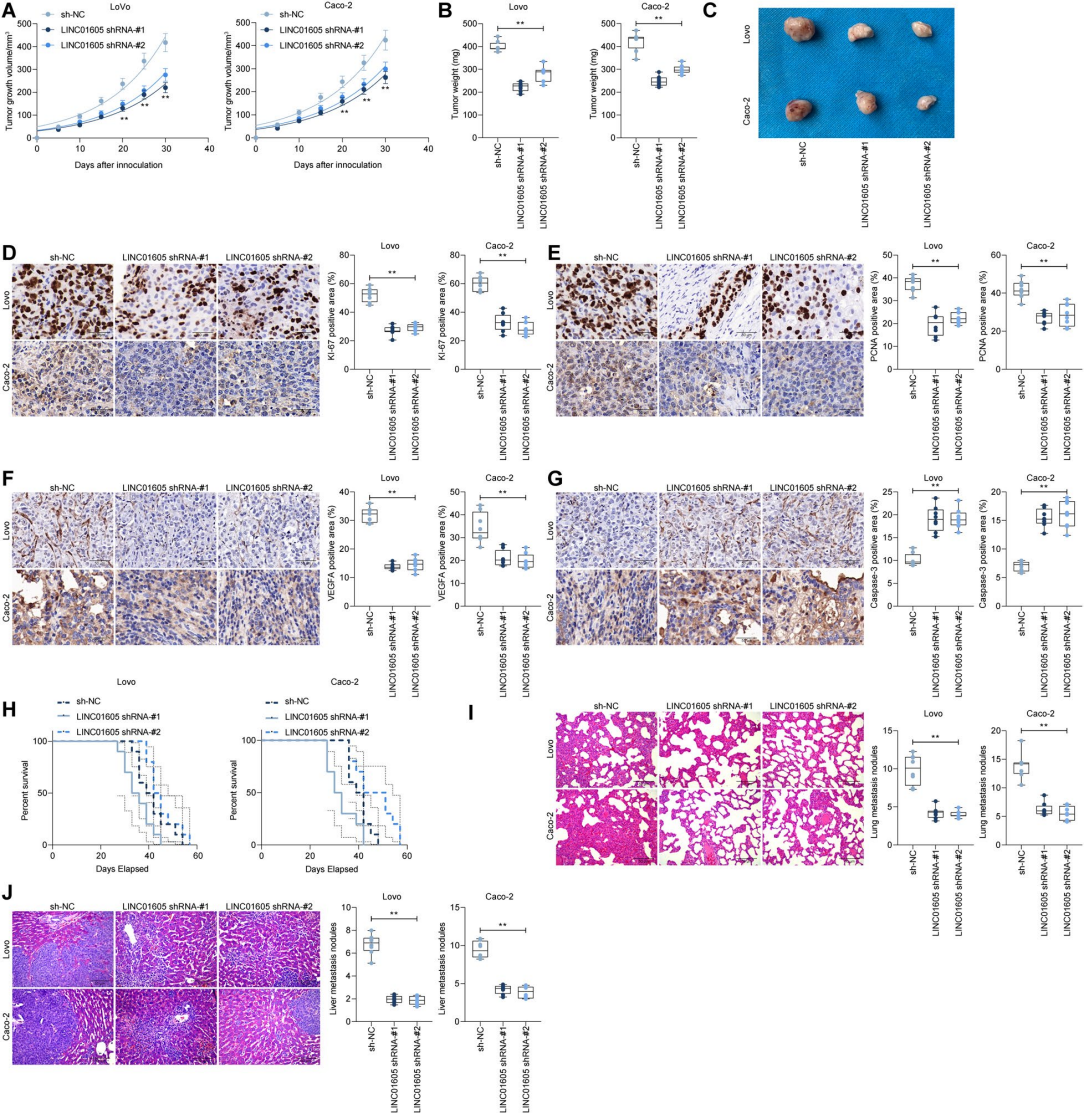
Knockdown of LINC01605 impairs the growth and metastatic activity of CC cells in vivo. LoVo and Caco-2 cells with stable low expression of LINC01605 were inoculated subcutaneously into NSG mice. **A** the tumor growth curves of each group of mice are summarized. **B** box plot for the average tumor weight. **C** representative images of xenograft tumors in nude mice after different treatments. **D**–**G**, immunohistochemical detection of KI67 (**D**), PCNA (**E**), VEGFA (**F**) and Caspase-3 (**G**) staining intensity in xenograft tumors formed by LoVo or Caco-2 cells. **H**, CC cells were injected intracardially into NSG mice, and the survival rate of NSG mice after cell inoculation was analyzed by Kaplan–Meier. **I**, **J**, 30 days after inoculation of CC cells, mice were euthanized, and the number of metastatic nodes was counted by extracting lung (**I**) and liver (**J**) tissues from mice. In **A**–**G**, **I** and **J**, each group contains eight mice; and in **H**, each group contains 10 mice, with each dot representing one mouse (**p < 0.01, one-way or two-way ANOVA, followed by Tukey’s multiple comparison)

To further clarify the role of LINC01605 in the growth of CC cells, we injected LoVo and Caco-2 cells with stable low expression of LINC01605 into NSG mice subcutaneously. Knockdown of LINC01605 drastically slowed down the growth of CC cells (Fig. 4A–C). Consequently, we assessed the staining intensity of KI67, PCNA, VEGFA, and Caspase-3 in the tumor tissues using immunohistochemistry. We observed that the staining intensity of KI67, PCNA, and VEGFA was significantly reduced, while the staining intensity of Caspase-3 was significantly increased in the tumors formed by CC cells with low expression of LINC01605 (Fig. 4D–G), indicating that knockdown of LINC01605 inhibited the growth of CC cells in vivo. In in vivo metastasis assays, we injected CC cells into NSG mice via intracardiac injection, and we found a significant increase in survival in mice administrated with LoVo cells and Caco-2 cells low in LINC01605 expression (Fig. 4H). Moreover, we collected lung and liver tissues from these mice. The lung and liver metastatic nodules formed by CC cells were reduced after knocking down LINC01605 (Fig. 4I, J). Combining the above experimental results, we can conclude that knocking down LINC01605 can significantly constrain the growth and metastasis of CC cells.

### Overexpression of SMYD2 or EP300 promotes malignant biological behaviors of CC cells low-expressing LINC01605

**Fig. 5:**
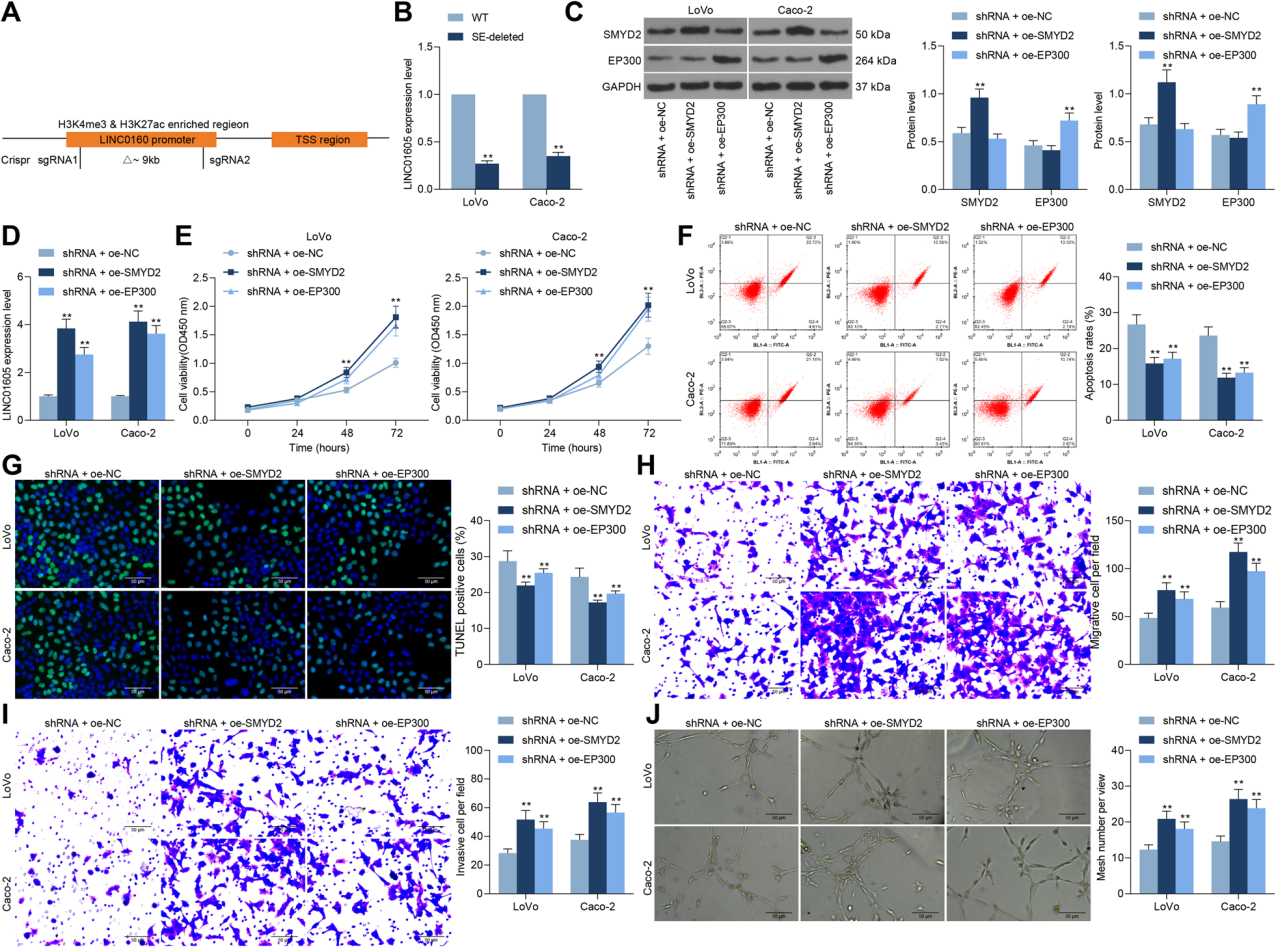
Overexpression of SMYD2 or EP300 promotes malignant biological behavior in CC cells. **A** schematic diagram of LoVo and Caco-2 cell lines with SE-deleted constructed by Crispr-Cas9 system. **B** detection of LINC01605 expression in SE-deleted LoVo and Caco-2 cells by RT-qPCR. Overexpression plasmids of SMYD2 or EP300 were transfected into cells with stable low expression of LINC01605. **C** SMYD2 or EP300 expression in cells after co-transfection measured using Western blot. **D** LINC01605 expression in LoVo and Caco-2 cells by RT-qPCR. **E** the proliferative activity of LoVo and Caco-2 examined using CCK-8 assay. **F** the proportion of apoptotic cells in LoVo and Caco-2 cells measured using flow cytometry analysis. **G** the number of apoptosis bodies in cells determined using TUNEL assay. **H**, **I**, the altered migration and invasion ability of LoVo and Caco-2 cells measured using Transwell assay. **J** the number of tubes formed by HUVEC cells co-cultured with Caco-2 and LoVo cells examined using tube formation assay. Error bars represent means ± SD for three independent experiments (**p < 0.01, two-way ANOVA, followed by Tukey’s multiple comparison)

Based on our previous experimental results, we hypothesized that SMYD2 and EP300 could promote the expression of LINC01605 through enhancer-like signature H3K27ac and H3K4me3, thus affecting colon carcinogenesis and progression. To verify the regulation of LINC01605 expression by H3K27ac or H3K4me3, we constructed SE-deleted LoVo and Caco-2 cell lines using the Crispr-Cas9 system (Fig. 5A). Moreover, RT-qPCR was conducted to validate that the expression of LINC01605 was drastically downregulated in the SE-deleted cells (Fig. 5B). Therefore, we transfected SMYD2 or EP300 overexpression plasmids into cells with stable low expression of LINC01605. The transfection efficiency was verified using western blot (Fig. 5C). We observed a significant increase in the expression of LINC01605 in cells after overexpression of SMYD2 or EP300 (Fig. 5D). We further found that increasing the expression of SMYD2 or EP300 in the cells also significantly enhanced the proliferative activity of the cells (Fig. 5E), which was accompanied by a significant decrease in the proportion of apoptotic cells and the number of apoptosis bodies (Fig. 5F, G). Subsequently, overexpression of SMYD2 or EP300 significantly enhanced the migration and invasion of LoVo and Caco-2 cells in Transwell assays (Fig. 5H, I). In tube formation assays, overexpression of SMYD2 or EP300 resulted in a significant augment in number of blood vessels (Fig. 5J).

### LINC01605 binds to METTL3 to promote the m6A modification of SPTBN2

**Fig. 6:**
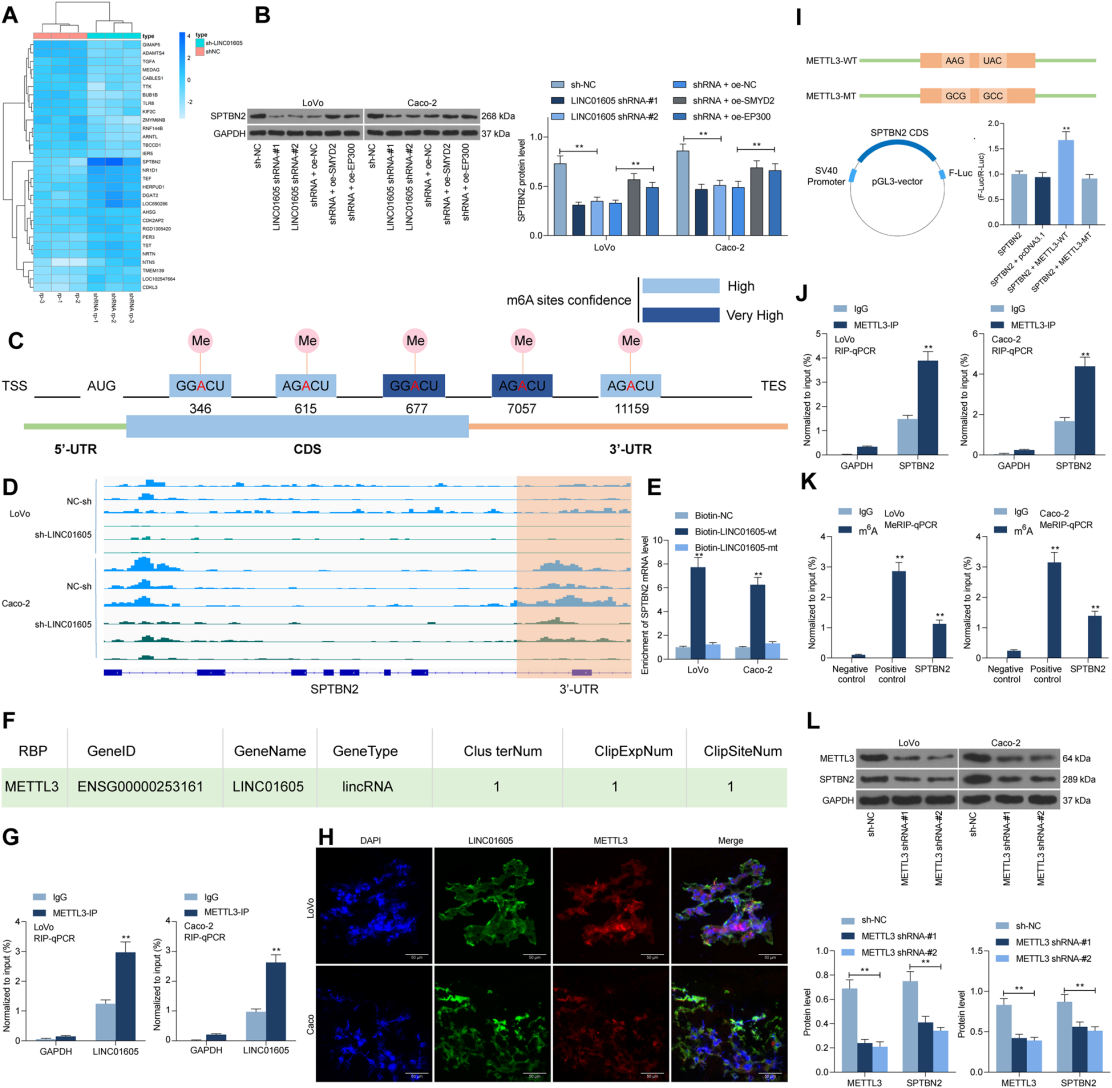
LINC01605 binds to METTL3 to promote the m6A modification of SPTBN2. **A** microarray analysis of genes differentially expressed in CC cells with knockdown of LINC01605. **B** SPTBN2 protein expression in LoVo and Caco-2 cells by western blot assay. **C** m6A modification sites of SPTBN2 mRNA predicted using SRAMP website. **D** m6A modification levels of SPTBN2 in LoVo and Caco-2 cells with sh-LINC01605 analyzed using MeRIP-seq. **E** the binding of LINC01605 to SPTBN2 mRNA verified using RNA pull-down assay. **F** regulatory-associated RBP bound to LINC01605 predicted using Starbase website. **G** proteins that bind to LINC1605 examined using RIP assays. **H** Cy3-label LINC01605 with anti-METTL3 for fluorescence co-localization assay to detect subcellular localization of LINC01605 with METTL3 in LoVo and Caco-2 cells. **I** diagrammatic sketch presented the construction of METTL3-WT and METTL3-MUT (up). diagrammatic sketch presented that the fragment of wild-type SPTBN2 CDS (SPTBN2) containing predicted METTL3 target sites was cloned into pGL3 vector with Firefly luciferase reporter genes (F-Luc) (down-left). The luciferase activity measurement in Lovo and Caco-2 cells (down-right). **J** the binding relationship between METTL3 and SPTBN2 mRNA measured using RIP assays. **K** detection of m6A modification levels of SPTBN2 mRNA in LoVo or Caco-2 cells by MeRIP-qPCR. **L** the shRNAs targeting METTL3 were transfected into LoVo or Caco-2 cells, and the protein expression of METTL3 and SPTBN2 in the cells were detected by western blot. Error bars represent means ± SD for three independent experiments (**p < 0.01, two-way ANOVA, followed by Tukey’s multiple comparison)

To further investigate the downstream mechanism of action of LINC01605, we used microarray sequencing to analyze the differentially expressed genes in CC cells with knockdown of LINC01605. Of them, SPTBN2 had a significant reduction after knockdown of LINC01605 (Fig. 6A). Western blot was then applied to examine the expression of SPTBN2 in LoVo and Caco-2 cells. The expression of SPTBN2 was significantly reduced after knockdown of LINC01605, but significantly increased after overexpression of SMYD2 or EP300 (Fig. 6B). Figure 6C exhibits a distinct m6A modification in the 3′-UTR sequence of SPTBN2 mRNA in the SRAMP website (http://www.cuilab.cn/sramp/). Moreover, we further analyzed the significantly reduced m6A modification of SPTBN2 in LoVo and Caco-2 cells with sh-LINC01605 using MeRIP-seq (Fig. 6D). With the help of RNA pull-down assays using Biotin-labeled LINC01605, we found that LINC01605 was able to enrich SPTBN2 mRNA (Fig. 6E). Thus, we speculated whether LINC01605 could bind to a particular m6A mediator, thus regulating the level of m6A modification of SPTBN2.

Therefore, the related RNA binding protein (RBP) bound by LINC01605 was predicted using the Starbase website (http://starbase.sysu.edu.cn/), which revealed that LINC01605 has a binding relationship with METTL3 (Fig. 6F). RIP further substantiated the binding between METTL3 and LINC01605 (Fig. 6G). We then performed fluorescence co-localization experiments using Cy3-label LINC01605 with anti-METTL3, and we found that LINC01605 and METTL3 had co-localization in LoVo and Caco-2 cells and localized in the cytoplasm (Fig. 6H). To further verify that METTL3 has a binding relationship with SPTBN2 mRNA, we first designed a pGL3 luciferase reporter vector containing the SPTBN2 CDS sequence and transfected it with METTL3-MT or WT into LoVo or Caco-2 cells. The results showed that METTL3-WT could increase luciferase activity in the cells (Fig. 6I). RIP-qPCR demonstrated the relationship between METTL3 and SPTBN2 mRNA, and we observed that in LoVo or Caco-2 cells, SPTBN2 RNA was enriched in METTL3-bound RNA (Fig. 6J). Moreover, to verify the regulatory effect of METTL3 on SPTBN2 mRNA, we transfected shRNAs targeting METTL3 in LoVo or Caco-2 cells. We observed a significant reduction in m6A modification of SPTBN2 mRNA in cells after knockdown of METTL3, and a significant decline in SPTBN2 protein expression as well (Fig. 6K, L). Combined with the above experimental results, it can be shown that LINC01605 can bind to METTL3 protein, which in turn promotes the m6A modification of SPTBN2 mRNA and enhances the translation of SPTBN2.

### LINC01605 harbors a positive correlation with SPTBN2 expression in CC patients

**Fig. 7:**
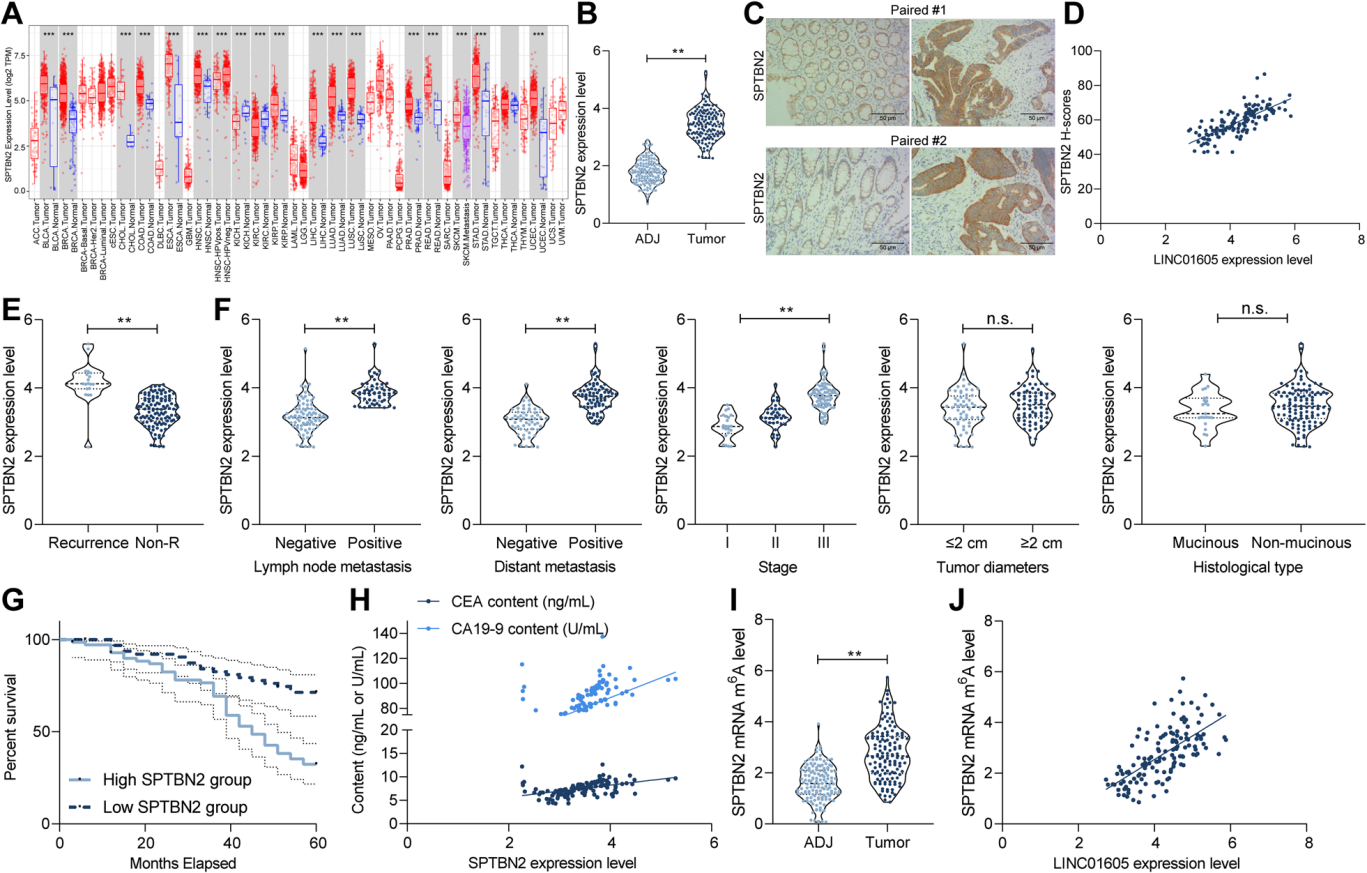
LINC01605 harbors a positive correlation with SPTBN2 expression in CC patients. **A** expression of SPTBN2 in multiple TCGA databases. **B** expression of SPTBN2 mRNA in tumor tissues and ADJ tissues of 134 CC patients by RT-qPCR. **C** immunohistochemistry staining of SPTBN2 in cancer and ADJ tissues of CC patients. **D** Spearman's correlation analysis of the staining intensity of SPTBN2 with the expression of LINC01605 in CC tissues. **E** SPTBN2 expression in tumor tissues of patients with or without recurrence by RT-qPCR. **F** the correlation between the expression of LINC01605 and the clinical phenotype of 134 patients. **G** Kaplan–Meier analysis of the correlation between SPTBN2 expression and survival in 134 patients with CC. **H** Spearman's correlation analysis of the expression of SPTBN2 with the levels of CEA and CA199, tumor markers of CC in patients’ serum. **I** analysis of m6A modification levels of SPTBN2 mRNA in tumor tissues and ADJ tissues by MeRIP-qPCR. **J** correlation between the m6A modification level of SPTBN2 mRNA and the expression of LINC01605. Error bars represent means ± SD for three independent experiments (**p < 0.01, paired or unpaired *t* test, one-way ANOVA, followed by Tukey’s multiple comparison)

To further validate the role of SPTBN2 in CC patients, we first analyzed the expression of SPTBN2 in TCGA-COAD database, and we found that SPTBN2 was significantly highly expressed in COAD patients and in a variety of cancers (Fig. 7A). Consistently, the expression of SPTBN2 was significantly elevated in our collected tumor tissues versus that in ADJ tissues (n = 134) (Fig. 7B). Moreover, the staining intensity of SPTBN2 in tumor tissues was positively correlated with the staining intensity of LINC01605 (Fig. 7C, D). In our next analysis, we found that the expression of SPTBN2 was also significantly higher in the tumor tissues of 19 patients with recurrence than in those without recurrence (Fig. 7E). More potential for lymph node metastases and advanced tumor stage was observed in CC patients with high expression of SPTBN2, but SPTBN2 expression was independent of the diameter and histological type of the tumor tissues (Fig. 7F). Moreover, patients with high SPTBN2 expression had a lower survival rate (Fig. 7G). We found that SPTBN2 was positively correlated with the levels of CC tumor markers CA199 and CEA in patients’ serum (Fig. 7H). Subsequently, we analyzed the m6A modification levels of SPTBN2 mRNA in tumor tissues and ADJ tissues using MeRIP-qPCR. The m6A modification of SPTBN2 was much higher in tumor tissues than that in ADJ tissues (Fig. 7I), which was positively correlated with the LINC01605 expression (Fig. 7J).

### SPTBN2 overexpression mitigates the inhibitory effects of LINC01605 silencing on CC cells

**Fig. 8:**
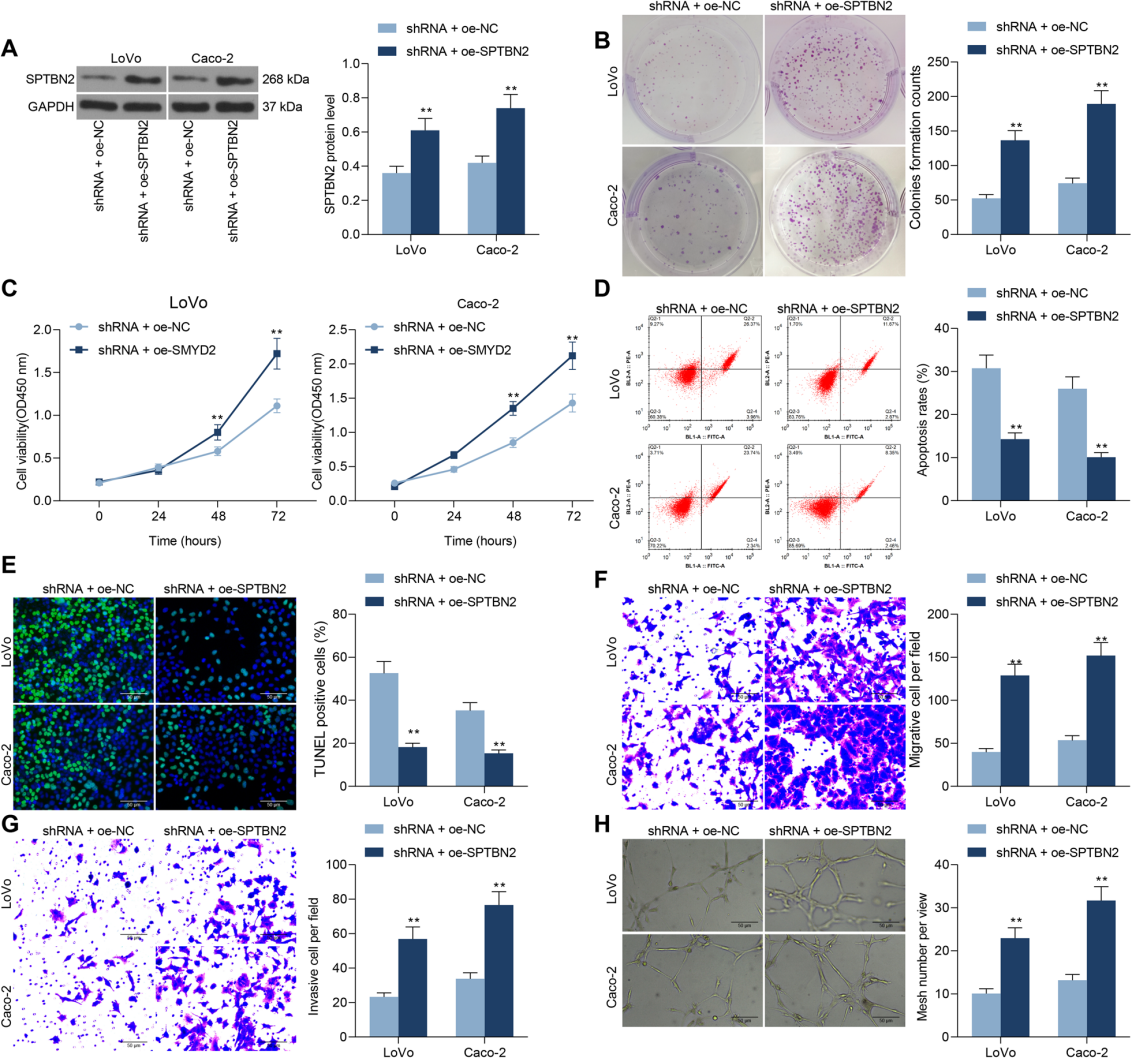
SPTBN2 overexpression reverses the repressive effects of LINC01605 silencing on CC cells. Overexpression plasmid of SPTBN2 was transfected into cells with stable low expression of LINC01605. **A** SPTBN2 protein expression in cells after co-transfection by Western blot. **B** the number of colonies formed by LoVo and Caco-2 examined using colony formation assay. **C** the proliferative activity of LoVo and Caco-2 examined using CCK-8 assay. **D** the proportion of apoptotic cells in LoVo and Caco-2 cells by flow cytometry. **E** the number of apoptosis bodies in cells by TUNEL assay. **F**, **G** the altered migration and invasion ability of LoVo and Caco-2 cells by Transwell assay. **H** the number of tubes formed by HUVEC cells co-cultured with Caco-2 and LoVo cells by tube formation assay. Error bars represent means ± SD for three independent experiments (**p < 0.01, two-way ANOVA, followed by Tukey’s multiple comparison)

To verify the effect of SPTBN2 on colon carcinogenesis and progression, we transfected the overexpression plasmid of SPTBN2 into cells with stable low expression of LINC01605. The transfection efficiency was determined using western blot (Fig. 8A). A significant increase in proliferative activity was observed in LoVo or Caco-2 cells after overexpression of SPTBN2 (Fig. 8B, C), which was accompanied by a significant decrease in the proportion of apoptotic cells and the number of apoptosis bodies (Fig. 8D, E). Subsequently, we found that overexpression of SPTBN2 significantly increased the migration and invasion of LoVo and Caco-2 cells in Transwell assays (Fig. 8F, G). After overexpression of SPTBN2, the number of tubes formed by LoVo and CaCo-2 cells was also significantly enhanced in the tube formation assay (Fig. 8H).

## Discussion

The occurrence of CRC, the most common and severe cancers worldwide, is developed by accumulation of genetic and epigenetic changes in colon cells [11]. It has been recently revealed that lncRNAs are implicated in many steps of cancer development through interactions with DNAs, RNAs, proteins and/or their combinations, serving as a critical driver in chromatin organization, and transcriptional and post-transcriptional mediations [12, 13]. In the current study, we discovered through analyzing five CC-related datasets from the GEO database and TCGA-COAD database that LINC01605 upregulation indicated poor prognosis in CC samples. We also presented that LINC01605 was overexpressed in CC tissues and cells and held prognostic significances in CC. Loss-of-function assays indicating that LINC01605 knockdown hampered the proliferation and metastases of CC cells, indicating that LINC01605 possessed oncogenic functions in CC.

To probe the upstream mechanism of LINC01605 in CC, we downloaded the ChIP-seq data from the GEO database for visualization. Combined with our findings derived from the immunohistochemistry and ChIP-qPCR, we corroborated that the overexpression of LINC01605 in CC was caused by the aberrant modification of H3K27ac and H3K4me3. Later bioinformatics prediction revealed that SMYD2 and EP300 was responsible for the aberrant modification of H3K4me3 and H3K27ac of LINC01605 in CC, respectively. SMYD2, a histone lysine methyltransferase that methylates H3K4 or H3K36, was significantly upregulated in ovarian clear cell carcinoma specimens compared with normal ovarian tissues [14]. In addition, it has been established to be closely linked to the clinicopathological features, including vascular invasion, TNM stage and lymph node metastasis of patients with CC [15]. Mechanistically, inhibition of SMYD2 suppressed the progression of renal cell carcinoma by downregulating microRNA-125b [16]. More relevantly, SMYD2 has been established to coordinate with EZH2 to promote breast cancer tumorigenesis and metastasis [17].

H3K27ac-induced lncRNA EIF3J-AS1 overexpression has been suggested to accelerate proliferation and impede apoptosis of CC cells [18]. As another major component of the complex, EP300 (also termed as KAT3B or p300) is predictive of worse prognosis in human malignancies, such as prostate, liver, kidney, and breast cancer [19]. Under the condition of oral squamous cell carcinoma, EP300 activated LINC00941 transcription through upregulating H3K27ac modification in its promoter [20]. In the present study, we provided evidence for the co-localization of SMYD2 and EP300 in the nucleus of CC tissues. Moreover, SE-depletion indeed led to the downregulation of LINC01605 in CC cell, further proving the overexpression of LINC01605 was SE-dependent SMYD2 and EP300 regulation. Later, overexpression plasmids of SMYD2 and EP300 successfully abrogated the anti-tumor effects of sh-LINC01605 in vitro, which corroborated our theory again.

After that, microarray sequencing was conducted on CC cells with sh-LINC01605 to figure out the downstream effector of LINC01605. SPTBN2 was noted to be the mostly downregulated one. Subsequently bioinformatics analysis revealed that there is a distinct m6A modification in the 3′-UTR sequence of SPTBN2 mRNA. The vital roles of m6A modification and its related regulatory proteins have been indicated in the pathogenesis of various malignancies, including gastric cancer [21], bladder cancer [22], breast cancer [23], lung cancer [24], as well as CC [25]. RNA pull-down and RIP assays in the present study attested the binding relation between LINC01605 and SPTBN2 or METTL3, respectively. pGL3 luciferase reporter vector, RIP-qPCR and western blot then confirmed the direct interaction between SPTBN2 and METTL3. Similar to our findings, METTL3-induced m6A modification was involved in the upregulation of CBX8, which contributed to increased cancer stemness and decreased chemosensitivity in CC [26]. The oncogenic role of SPTBN2 has been rarely investigated, whereas our prognostic analysis demonstrated that patients with higher expression of SPTBN2 suffered from short survival and more advanced tumor stage, which was correlated with its m6A modification. Further rescue experiments also validated that overexpression of SPTBN2 led to potentiated CC cell proliferation, migration, invasion, and tube formation.

## Conclusions

**Fig. 9:**
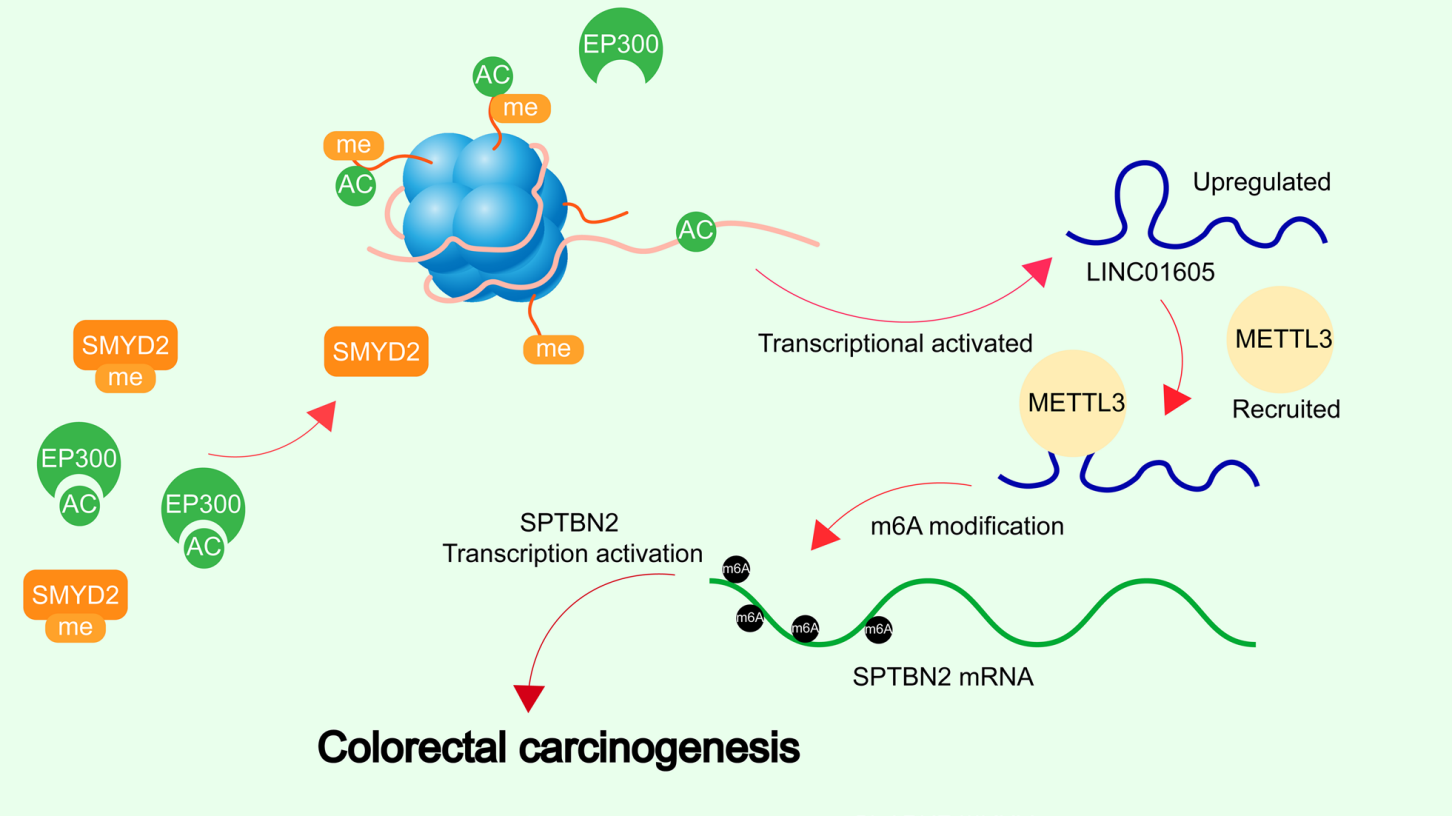
The schematic figure. High expression of LINC01605, regulated by SMYD2/EP300-mediated H3K4me3 and H3K27ac modifications in CC, binds to METTL3 protein to promote the m6A modification of SPTBN2 mRNA and the translation of SPTBN2, which in turn leads to the development of CC

In summary, our current work showed that LINC01605 was upregulated in CC and that LINC01605 high expression was associated with poor survival of patients with CC. Moreover, this study delineated that SMYD2/EP300-induced LINC01605 promoted proliferation, migration and invasion, and impeded apoptosis of CC cells through METTL3/SPTBN2 axis (Fig. 9). The discovery of this axis and its impact on CC metastasis will aid in further CC study and in exploring effective therapeutic options against CC.

## Supplementary Information

**Additional file 1:** Additional material and methods.

**Additional file 2:Figure S1.** The expression and prognostic value of ZNF337-AS1, LINC01082, DICER1-AS1, LUCAT1, MEG9, and SNHG20 in CC. A, The expression of ZNF337-AS1, LINC01082, DICER1-AS1, LUCAT1, MEG9, and SNHG20 in cancer and ADJ tissues of 134 CC patients were detected by RT-qPCR. B, Expression of ZNF337-AS1, LINC01082, DICER1-AS1, LUCAT1, MEG9, and SNHG20 in the TCGA-COAD database. C, Kaplan–Meier analysis of the correlation between the expression of ZNF337-AS1, LINC01082, DICER1-AS1, LUCAT1, MEG9, SNHG20 and the survival of COAD patients. Error bars represent means ± SD for three independent experiments (*p < 0.05 and ***p < 0.001. Paired or unpaired t test).

**Additional file 3: Figure S2.** LINC01605 is regulated by the SMYD2-EP300 complex. A, cis-regulatory elements upstream of LINC01605 predicted using UCSC website. B-C, ChIP-seq data (GSE36204) of normal colon tissue versus CC tissue were downloaded, and Bowtie2 analyzed the peaks of H3K4me1, H3K4me3, and H3K27ac.

## Data Availability

The datasets used and analyzed in the current study are available from the corresponding author in response to reasonable requests.

## Abbreviations

CC: Colorectal cancer
CCK-8: Cell counting kit-8
ChIP: Chromatin immunoprecipitation
COAD: Colon adenocarcinoma
CRISPR: Clustered regularly interspaced short palindromic repeats
DAPI: 4′,6-Diamidino-2-phenylindole
FITC: Fluorescein isothiocyanate
GAPDH: Glyceraldehyde-3-phosphate dehydrogenase
GEO: Gene expression omnibus
lncRNAs: Long non-coding RNAs
MeRIP: Methylated RNA immunoprecipitation sequencing
NC: Negative control
NGS: Next generation sequencing
OD: Optical density
RIP: RNA-immunoprecipitation
RT-qPCR: Reverse transcription-quantitative PCR
seq: Sequencing
sgRNAs: Single-guide RNAs
TUNEL: Terminal deoxynucleotidyl transferase (TdT)-mediated 2′-deoxyuridine 5′-triphosphate (dUTP) nick end labeling
