# Supplementary material for: LINC01605, regulated by the EP300-SMYD2 complex, potentiates the binding between METTL3 and SPTBN2 in colorectal cancer
Source: Cancer Cell Int. 2021 Sep 20;21:504. doi: 10.1186/s12935-021-02180-8 (PMC8451128; doi:10.1186/s12935-021-02180-8)
Supplement: Supplementary file 1 — Additional file 1: Additional material and methods. [file 12935_2021_2180_MOESM1_ESM.docx]

**Reverse transcription-quantitative PCR (RT-qPCR)**

Total RNA was isolated from tissues or Lovo and Caco-2 cells using RNAzol reagent (Sigma-Aldrich, St. Louis, MO, USA). Total RNA was reversely transcribed into cDNA using the GoScript™ Reverse Transcription System (Promega Corporation, Madison, WI, USA). The reverse transcription reaction was carried out at 42˚C for 15 min, followed by 95˚C for 3 min. To detect the expression of the genes of interest, qPCR reaction mixtures were prepared using SYBR Green Master Mix (Bio-Rad Laboratories, Hercules, CA, USA). Expression of target genes were normalized to the expression of glyceraldehyde-3-phosphate dehydrogenase (GAPDH). The thermocycling conditions were as follows: initial denaturation at 95˚C for 2 min, followed by 40 cycles of amplification at 95˚C for 10 s and 60˚C for 32 s. The relative transcription level was calculated with 2^-∆∆ct^. The primers of the following transcripts were LINC01605 forward: 5’-CAGCCACAAGGAAAAGGCAC-3’; reverse 5’-TGCCAAGAAGCTGTCCTCTG-3’; ZNF337-AS1 forward: 5’-CTGCATGTCTTAGGGGTGGG-3’; reverse: 5’-CTGCATGTCTTAGGGGTGGG-3’; LINC01082 forward: 5’-CGGACTCTATCGAGGCACAC-3’; reverse: 5’-TCCGCTCTTGGTGGTCTTTC-3’; DICER1-AS1 forward: 5’-CTTGGTAGTAGGATTGCTGGATC-3’; reverse: 5’-GGCAGAGGTTACAGTGAGCCAA-3’; LUCAT1 forward: 5’-TTATGCAACGCCAGGTGCTA-3’; reverse: 5’-GGGGTAGAGGGTACAGTGGT-3’; MEG9 forward: 5’-CGACGATGGGATAGGCAGAC-3’; reverse: 5’-TCTGGATGGTTTCCTGCACC-3’; SNHG20 forward: 5’-GATACACGCCAAGGTGACCA-3’; reverse: 5’-GATACACGCCAAGGTGACCA-3’; EP300 forward: 5’-GATGACCCTTCCCAGCCTCAAA-3’; reverse: 5’-GCCAGATGATCTCATGGTGAAGG-3’; SMYD2 forward: 5’-AAGGCAGAAGCCATCCGAGACA-3’; reverse: 5’-TCATCTTCTCCTGGCTGAGCTC-3’; SPTBN2 forward: 5’-AGTGGCAGAAGCACCAGGCATT-3’; reverse: 5’-TTCTCCGACACCAGGGCTTTCA-3’; METTL3 forward: 5’-CTATCTCCTGGCACTCGCAAGA-3’; reverse: 5’-GCTTGAACCGTGCAACCACATC-3’; GAPDH forward: 5’-GTCTCCTCTGACTTCAACAGCG-3’; reverse: 5’-ACCACCCTGTTGCTGTAGCCAA-3’.

**Western blot**

Proteins were isolated from cells using radio immunoprecipitation assay lysis buffer containing phenylmethanesulfonyl fluoride (Thermo Fisher Scientific Inc., Waltham, MA, USA), and protein concentrations were determined using a bicinchoninic acid assay protein assay kit (Beyotime, Shanghai, China). Protein extracts were run on 12% SDS-PAGE, and the separated proteins (~50 µg) were transferred to polyvinylidene difluoride membranes. Non-specific binding was blocked by 5% skimmed milk in TBS-0.1% Tween-20 buffer. The membranes were incubated with antibodies against SPTBN2 (1:500, ab92307, Abcam, Cambridge, UK) and GAPDH (1:500, ab9484, Abcam) at 4℃. The membranes were re-probed for 60 min at room temperature using HRP-conjugated secondary antibodies, and images were finally obtained using a BioSpectrum 600 imaging system (UVP, Inc., Upland, CA, USA).

**Immunohistochemistry**

Paraffin-embedded sections were deparaffinized, rehydrated, and treated with 3% hydrogen peroxide for 10 min. The antigen was retrieved by boiling in citrate solution (pH = 6.0) for 2 min. After that, the sections were cultured in 5% bovine serum albumin for 0.5 h and incubated with primary antibodies to KI67 (#sc-23900, Santa Cruz Biotechnology Inc., Santa Cruz, CA, USA) and SPTBN2 (#sc-376487, Santa Cruz Biotechnology) overnight at 4°C. Goat anti-mouse secondary antibody conjugated with horseradish peroxidase was used for a 30-min incubation at room temperature. A complex of streptavidin-peroxidase and 3,3′-diaminobenzidine substrate was then applied for color development. The slides were counter-stained with hematoxylin and photographed under a light microscope (BX51, Olympus, Tokyo, Japan). The slides were analyzed under a light microscope. For each field, the intensity (weak, medium, strong) and percentage of positively stained cells were quantified. The semi-quantitative H-score was calculated as: percentage of weakly staining cells + percentage of moderately staining cells × 2 + percentage of strongly staining cells × 3. The final scores range from 0 to 300. The H-score analysis was performed independently by two pathologists.

**Colony formation assay**

After being cultured in 6-well plates (1 × 10^3^ cells/well) for 3 weeks, the cells were subjected to a 15-min methanol fixation and a 1-h 2% crystal violet staining. Images of the colonies were captured with a ChemiDoc imaging system (Bio-Rad), and then the colonies were counted using Image J software.

**Cell counting kit-8 (CCK-8)**

At 24 h post-transfection, cell proliferation assays were performed using CCK-8 (ab228554, Abcam). CCK-8 solution (10 μL) was added at 24, 48, 72 and 96 h after the start of cell culture. After that, the cells were incubated for another 4 h, and 10 μL of dimethyl sulfoxide was added. The optical density (OD) value at 450 nm was measured to reflect the cell proliferation capacity.

**Flow cytometry**

An Annexin V-fluorescein isothiocyanate (FITC) Apoptosis Detection Kit (Affymetrix, Santa Clara, CA, USA) was used to analyze the apoptosis of CC cells. The cells analyzed with a FACS flow cytometer (BD Biosciences, San Jose, CA, USA).

**TUNEL assay**

Apoptotic cells were evaluated according to the instructions of the TUNEL Apoptosis Detection Kit (Beyotime). Briefly, transfected CC cells (1 × 10^5^) were fixed with 4% paraformaldehyde (p1110, Solarbio, Beijing, China) at 4ºC for 30 min and treated with 0.3% TritonX-100 (9002-93-1, Sigma-Aldrich) for 5 min. Afterward, the TUNEL solution (c1088, Beyotime) was replenished to each well for a 60-min incubation at 37ºC in the dark. After that, the CC cells were stained with 4',6-diamidino-2-phenylindole (DAPI, Sigma-Aldrich) for 10 min at room temperature in the dark, and then observed and photographed under an inverted fluorescence microscope. Finally, the positive cell rate was counted using Image-J software and defined as TUNEL-positive cells/DAPI-positive cells.

**Transwell assay**

Transwell migration and invasion assays were carried out to assess the cell migratory and invasive abilities. Transwell chambers (pore size, 6.5 mm) were put into 24-well plates, 1 x 10^5^ cells resuspended in 200 µL serum-free DMEM were seeded into the apical chamber (Corning Glass Works, Corning, N.Y., USA) without (transwell migration assay) or with (transwell invasion assay) Matrigel. While 600 µL DMEM with 10% FBS was added into the basolateral chamber. After 24 h, the cells in the apical chamber were removed using a cotton swab, and the cells on the surface of the basolateral chamber membrane were fixed by 4% paraformaldehyde for 15 min and stained with 0.1% crystal violet for 20 min at room temperature. Image acquiring and cells counting were performed with a BX51 light microscope (Olympus Corporation) at magnification of x200 in eight randomly selected fields.

**Tube formation assay**

The 96-well plates were coated with Matrigel (BD Biosciences; 100 μL/well) and subsequently left to stand for 30 min at 37ºC. After that, human umbilical vein endothelial cells (HUVECs) were seeded into the plates at 1 × 10^4^ cells/well. After the cells have fully adhered to the wells, the medium was replaced with conditioned medium for Caco-2 and LoVo cells. After 24 h of culture. The cells were monitored and imaged using an Olympus DP71 immunofluorescence microscope (Olympus), and the number of tubes formed in the focal region were measured and analyzed using Chemi Imager 5500 V2.03 software (Alpha Innotech, San Leandro, CA, USA).

***In vivo* tumor formation**

A total of 156 immunodeficient humanized NOD-scid IL-2Rγ^(-/-)^ (NSG) mice were from Vital River (Beijing, China). For the tumor xenograft model, 8 mice were injected subcutaneously with 5 × 10^6^ LoVo or Caco-2 cells stably transfected with LINC01605 shRNA-#1, LINC01605 shRNA-#2 or sh-NC on the right flank. The tumor volume (length × width^2^/2) was recorded every 5 days, and the mice were euthanized after 30 d.

***In vivo* metastasis assay**

For the *in vivo* metastasis assay, 2 × 10^6^ LoVo or Caco-2 cells stably transfected with LINC01605 shRNA-#1, LINC01605 shRNA-#2 or sh-NC were injected intracardially into 8 mice, and the mice were euthanized after 35 d. Liver tissue and lung tissue from mice were collected for detection of the number of metastatic nodules. Another 10 NSG mice from each group were taken and injected with CC cells to test the survival rate of mice.
