# Supplementary figures and images for: LINC01605, regulated by the EP300-SMYD2 complex, potentiates the binding between METTL3 and SPTBN2 in colorectal cancer
Source: Cancer Cell Int. 2021 Sep 20;21:504. doi: 10.1186/s12935-021-02180-8 (PMC8451128; doi:10.1186/s12935-021-02180-8)

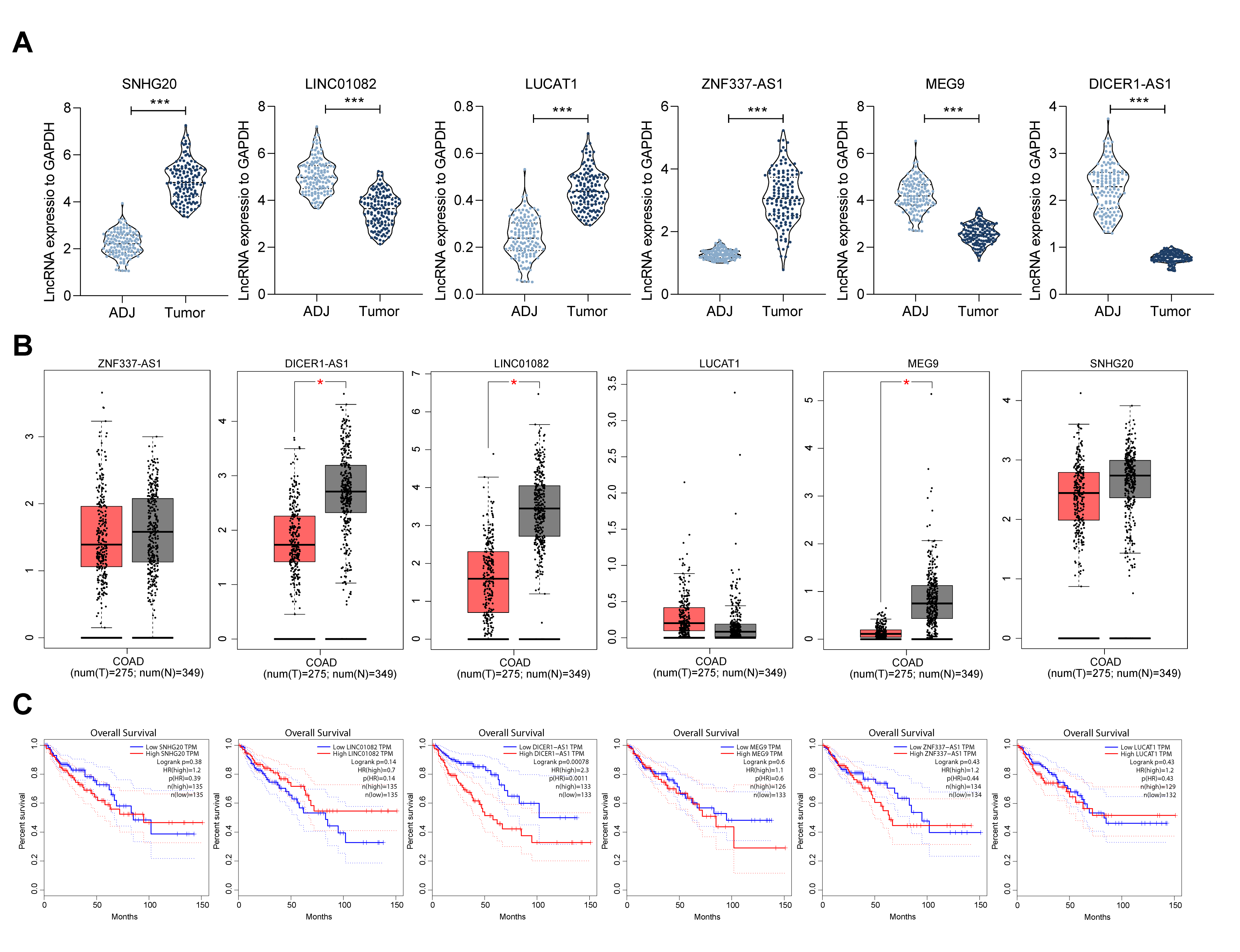

Supplement: Supplementary file 2 — Additional file 2:Figure S1. The expression and prognostic value of ZNF337-AS1, LINC01082, DICER1-AS1, LUCAT1, MEG9, and SNHG20 in CC. A, The expression of ZNF337-AS1, LINC01082, DICER1-AS1, LUCAT1, MEG9, and SNHG20 in cancer and ADJ tissues of 134 CC patients were detected by RT-qPCR. B, Expression of ZNF337-AS1, LINC01082, DICER1-AS1, LUCAT1, MEG9, and SNHG20 in the TCGA-COAD database. C, Kaplan–Meier analysis of the correlation between the expression of ZNF337-AS1, LINC01082, DICER1-AS1, LUCAT1, MEG9, SNHG20 and the survival of COAD patients. Error bars represent means ± SD for three independent experiments (*p < 0.05 and ***p < 0.001. Paired or unpaired t test). [file 12935_2021_2180_MOESM2_ESM.tif]

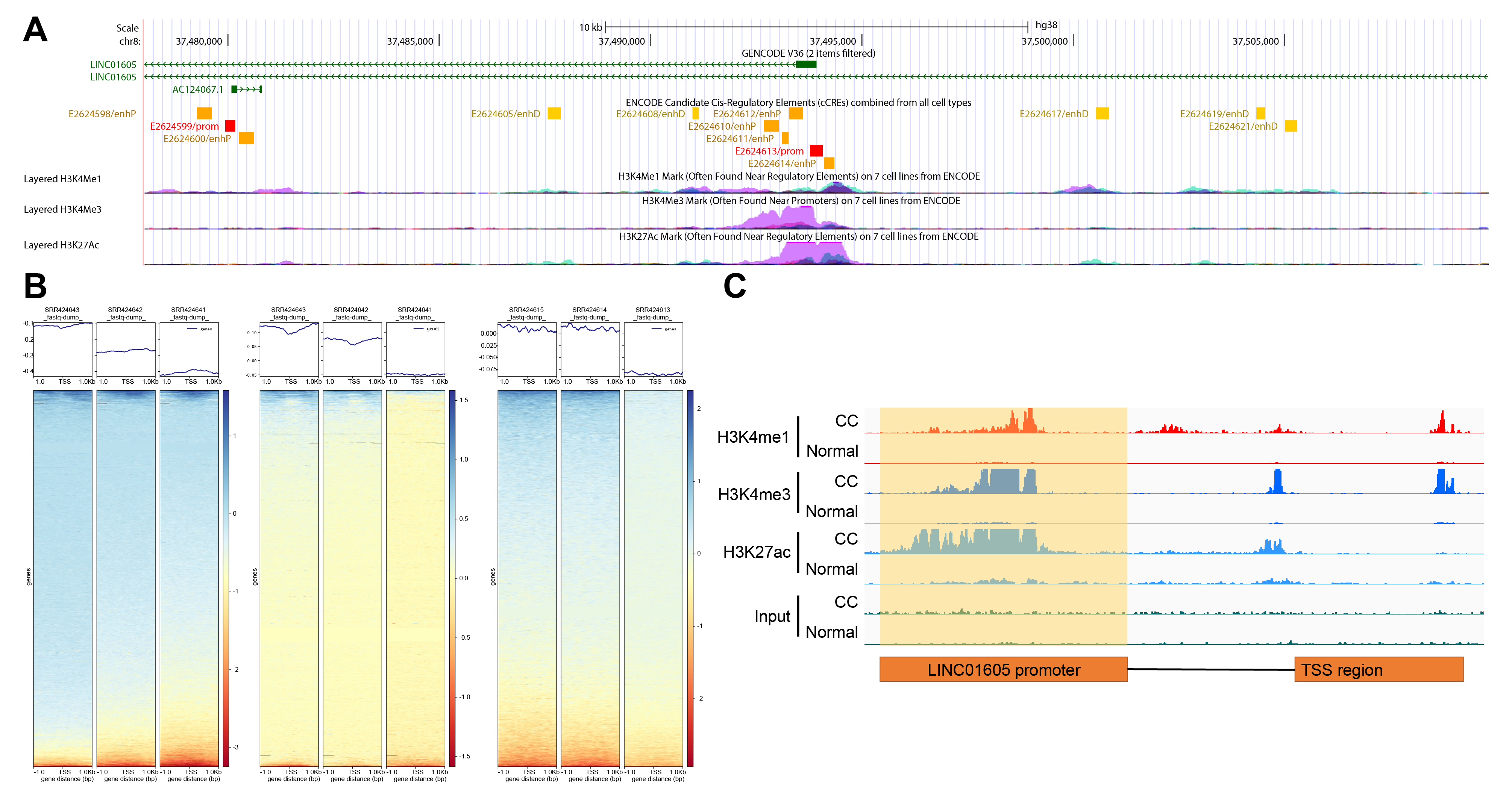

Supplement: Supplementary file 3 — Additional file 3: Figure S2. LINC01605 is regulated by the SMYD2-EP300 complex. A, cis-regulatory elements upstream of LINC01605 predicted using UCSC website. B-C, ChIP-seq data (GSE36204) of normal colon tissue versus CC tissue were downloaded, and Bowtie2 analyzed the peaks of H3K4me1, H3K4me3, and H3K27ac. [file 12935_2021_2180_MOESM3_ESM.tif]
